# High Seropositivity of *Brucella melitensis* Antibodies among Pregnant Women Attending Health Care Facilities in Mwanza, Tanzania: A Cross-Sectional Study

**DOI:** 10.1155/2023/2797441

**Published:** 2023-08-26

**Authors:** Helmut A. Nyawale, Michael Simchimba, Joseph Mlekwa, Fridolin Mujuni, Elieza Chibwe, Prosper Shayo, Elifuraha B. Mngumi, Khadija S. Majid, Mtebe Majigo, Stephen E. Mshana, Mariam M. Mirambo

**Affiliations:** ^1^Department of Microbiology and Immunology, Weill Bugando School of Medicine, Catholic University of Health and Allied Sciences, P.O. Box 1464, Mwanza, Tanzania; ^2^Department of Obstetrics and Gynecology, Weill Bugando School of Medicine, Catholic University of Health and Allied Sciences, P.O. Box 1464, Mwanza, Tanzania; ^3^Department of Veterinary Pathology, Sokoine University of Agriculture, P.O. Box 3018, Morogoro, Tanzania; ^4^Department of Veterinary Medicine and Public Health, Sokoine University of Agriculture, Morogoro P.O. Box 3021, Tanzania; ^5^Department of Microbiology and Immunology, Muhimbili University of Health and Allied Sciences, P.O. Box 65001, Dar es Salaam, Tanzania

## Abstract

**Background:**

Brucellosis is one of the most prevalent zoonotic neglected tropical diseases across the globe. *Brucella melitensis* (*B. melitensis*), the most pathogenic species is responsible for several pregnancy adverse outcomes in both humans and animals. Here, we present the data on the magnitude of *B. melitensis* antibodies among pregnant women in Mwanza, Tanzania, the information that might be useful in understanding the epidemiology of the disease and devising appropriate control interventions in this region. *Methodology*. A hospital-based cross-sectional study involving pregnant women was conducted at two antenatal clinics in Mwanza between May and July 2019. The pretested structured questionnaire was used for data collection. Blood samples were collected aseptically from all consenting women followed by the detection of *B. melitensis* antibodies using slide agglutination test. Descriptive data analysis was done using STATA version 17.

**Results:**

A total of 635 pregnant women were enrolled with the median age of 25 (interquartile range (IQR): 16-48) years and median gestation age of 21 (IQR: 3-39) weeks. Seropositivity of *B. melitensis* antibodies was 103 (16.2 (95% CI:13.3-19.1)). On the multivariate logistic regression analysis, as the gestation age increases, the odds of being seropositive decreases (aOR:0.972 (95% CI: 0.945-0.999), *P* = 0.045). Furthermore, being a housewife (aOR:3.902 (95% CI:1.589-9.577), *P* = 0.003), being employed (aOR:3.405 (95% CI:1.412-8.208), *P* = 0.006), and having history of miscarriage (aOR:1.940 (95% CI:1.043-3.606), *P* = 0.036) independently predicted *B. melitensis* seropositivity among pregnant women in Mwanza.

**Conclusion:**

High seropositivity of *B. melitensis* was observed among employed and housewife pregnant women in Mwanza. This calls for the need of more studies in endemic areas that might lead to evidence-based control interventions.

## 1. Background

Brucellosis caused by different *Brucella* species (*Brucella* spp.) is one of the most prevalent zoonotic diseases affecting both humans and animals with high prevalence reported in tropical regions [1]. *Brucella* spp. infections have been well known to cause abortions and other adverse pregnancy outcomes mainly in ruminants [1, 2]. The disease is common in different parts of the world including sub-Saharan Africa, Middle East, Central Asia, South America, and Eastern Europe [3–6]. The incidence of human brucellosis has been found to range from <0.01 to >200 per 100,000 population [7]. Nevertheless, the incidence among pregnant women in endemic areas has been found to range from 1.3% to 12.2% [8–10].

Brucella infections play an important role in women's health due to its devastating effects in causing adverse pregnancy outcomes. Brucellosis has been linked with preterm delivery, low birth weight, spontaneous abortions, and fetal deaths [11–15]. The symptoms of brucellosis resemble those of other tropical diseases including malaria, typhoid fever, and leptospirosis, making clinical diagnosis difficult [13, 16]. Nevertheless, it is not included in the routine diagnosis in many centers in low- and middle-income countries (LMICs), making it one of the underdiagnosed diseases in most of these countries. Moreover, this poses challenges in management and estimating a true disease burden. Several factors have been linked to *Brucella* spp. infections including consumption of raw/unpasteurized milk and its products, consumption of raw/undercooked meat especially grilled meat, contact with animal fluids, and working in abattoirs/handling animals without wearing protective gear [2, 17, 18].

The primary host of *B. melitensis* is a goat; however, it has a potential to infect a wide range of hosts including human. A previous study reported a significant contamination of sheep, cattle, and goat meat with *Brucella* spp. indicating the possible risk of transmission among meat handlers and consumers [19]. Moreover, goat meat is commonly consumed as grilled meat in urban settings of the tropics due to its nutritive values [20] making goats as the main source of Brucella infections among individuals preparing and consuming goat meat in these settings.

Despite having a wide range of hosts, *B. melitensis* is commonly known to cause clinical and subclinical infections in human as well as severe forms of the disease in both human and animals compared to other species. Despite being the most pathogenic among all species, its magnitude among human population is not well established [21]. Understanding *B. melitensis* epidemiology is of paramount importance in devising evidence-based control interventions using one health approach. This study documents the seropositivity of *B. melitensis* and associated factors among pregnant women attending health care facilities in Mwanza, Tanzania.

## 2. Methodology

### 2.1. Study Design, Site, and Study Duration

A hospital-based cross-sectional study was conducted between May and July 2019 among pregnant women attending antenatal clinics in the city of Mwanza, Tanzania. The study was conducted at Makongoro Health Center (MHC) and Sengerema Designated District Hospital (SDDH) antenatal clinics. SDDH has 35 wards with a bed capacity of 320 serving a catchment population of 663,034. SDDH attends an average of 35 to 40 pregnant women per day with approximately 700 pregnant women being attended monthly. MHC is located in Nyamagana district and is serving a population of about 1,090 pregnant women per month with an average of 80 to 100 pregnant women being attended per day.

### 2.2. Study Population, Sample Size Estimation, and Sampling Procedures

This study included all pregnant women regardless of the gestation age attending antenatal care at SDDH and MHC during the study period. The sample size was estimated using the Kish Leslie formula using the prevalence of 50%. The estimated sample size was 384 with a design effect of 1.65, making a sample size of 635. A total of 635 pregnant women were serially recruited until the sample size was reached.

### 2.3. Data and Specimen Collection

A pretested structured questionnaire was used to collect sociodemographic, clinical, and other relevant information. A 5 ml of venous blood was collected by a qualified phlebotomist in a plain vacutainer tube (Becton & Dickson Co. Ltd., Nairobi, Kenya). The collected specimens were labelled with a participant's unique identification number. Sera were separated from the whole blood by centrifugation at 3000 rpm for 4 min. All sera were stored at -80°C until processing.

### 2.4. Laboratory Procedures

Sera were removed from a deep freezer and left at room temperature for 20 to 30 minutes in order to allow thawing then analyzed using slide agglutination test as per manufactures instructions (Euromedi equip Ltd., UK). The test has been found to have a sensitivity and specificity of 95% and 100%, respectively [22]. Reagents and sample were brought to room temperature; then, 50 *μ*l of sample and 1 drop of each control (positive and negative) were placed into separate circles on the slide. Then, the antigen vial was swirled gently before use. One drop (50 *μ*l) of antigen was added to each circle, followed by gentle mixing. The slide was then manually rotated for 2 min and inspected. The results were read immediately by noting the agglutination when visible. In case of unclear results, the second person was asked to read the slide and results compared. The known positive and negative control samples were run along with test samples, and the results were compared to each other.

### 2.5. Data Analysis

Data was cleaned, coded, and analyzed using STATA version 17 (StataCorp LLC). Categorical variables were summarized as frequencies and proportions while continuous variables were summarized as median with interquartile ranges (IQR). Logistic regression analysis was performed for all variables known to be associated with Brucellosis. Socioeconomic status (SES) was determined by using source of water, toilet type, and house type. All variables with *P* value of less than 0.05 were subjected into multivariate logistic regression analysis adjusted by age to determine the association between predictor variables and the outcome. Level of education was not subjected on the multivariate analysis because of its collinearity with occupation. A *P* value of less than 0.05 was considered as statistically significant.

## 3. Results

### 3.1. Sociodemographic Characteristics of the Enrolled Study Participants (*N* = 635)

A total of 635 pregnant women were enrolled and included in the final analysis of this study. The median age of study participants was 25 (IQR: 16-48) years. The median gestation age was 21 (IQR:3-39) weeks while the median parity was 1 (IQR: 1-18) children. More than half of the enrolled women 359 (56.5%) were married while the majority 384(60.5%) were from urban areas. About a quarter of 150 (23.6%) of the participants reported a history of keeping animals at home while more than three quarters 507 (79.8%) were found to have high socioeconomic status (SES) (Table 1).

### 3.2. Clinical Characteristics of the Participants (*N* = 635)

The median body temperature at enrollment was 36.3°C (IQR:35.5-36.7); however, 306 (48.2%) of the participants reported to have fever in the current pregnancy. More than one-third 233 (36.7%) reported to have malaise in the current pregnancy while half of them 328 (51.7%) reported to experience headache in the current pregnancy. Almost one-third 204 (32.1%) reported to have loss of appetite in the current pregnancy while 63 (9.9%) of them experienced myalgia.

Regarding eating habits, 26 (4.1%) reported to consume raw meat while 405 (63.8%) reported to consume roasted meat. Regarding outcomes of previous pregnancies, 26 (5.8%), 72 (11.3%), and 12 (2.7%) reported to have babies with low birth weight, miscarriage, and stillbirth, respectively. History of blood transfusion was reported in 47 (7.4%) while 12 (1.9%) were HIV seropositive (Table 2).

### 3.3. Seropositivity of *B. Melitensis* and Associated Factors among Pregnant Women in Mwanza (N =635)

Out of 635 enrolled pregnant women, 103 (16.2% (95% CI:13.3-19.1)) were found to be *B. melitensis* antibodies, seropositive. On the multivariate logistic regression analysis, as the gestation age increases, the odds of being seropositive decrease (aOR:0.972, 95% CI: 0.945-0.999, *P* = 0.045). Furthermore, being housewife (aOR:3.902, 95% CI:1.589-9.577, *P* = 0.003), being employed (aOR:3.405, 95% CI:1.412-8.208, *P* = 0.006), and having history of miscarriage (aOR:1.940,95% CI:1.043-3.606, *P* = 0.036) independently predicted *B. melitensis* seropositivity (Table 3).

## 4. Discussion

Brucellosis is a common zoonotic disease affecting human livelihood mostly in tropical regions. Control efforts can be successful if the magnitude and epidemiology of the diseases are well understood. This study is aimed at determining the magnitude of *B. melitensis* antibodies among pregnant women in Mwanza, Tanzania. We observed that 16.2% of pregnant women had *B. melitensis* antibodies. We also observed various factors such as being a housewife, employed, and gestational age to be associated by the presence of *B. melitensis* antibodies. The information obtained is useful in the design of the control strategies of Brucella infections among pregnant women in endemic areas such as the study setting.

Globally, the seroprevalence of Brucellosis has been reported to range from 1.5 to 12.2% [23]. However, in this study, seropositivity of *B. melitensis* was found to be 16.2% which was high compared to a previous report in Katavi which documented seropositivity of 10.9% [24]. This could be explained by the fact that there are more livestock activities and human-to-human interactions in Mwanza than Katavi that might significantly make pregnant women in Mwanza more exposed than those in Katavi. In comparison to previous studies in Mwanza among livestock keepers and pregnant women which reported seropositivity of 11% and 7.6%, respectively [25, 26], the seropositivity reported in the current study is significantly high. This could be explained by possible epidemiological changes that have occurred in the past eight years.

In comparison to previous studies in Pakistan and Thailand among pregnant women residing in urban areas which reported seropositivity of 5.8% and 3.7%, respectively [27, 28], the reported seropositivity in the current study is significantly high. This could partly be explained by differences in geographical locations and the diagnostic test used; in these studies, enzyme-linked immunosorbent assay (ELISA) techniques were used, contrary to the current study that used a slide agglutination test. ELISA technique has been found to have high specificity compared to agglutination test.

Among the factors assessed in this study, as gestation age (GA) increases, the likelihood of being seropositive decreases. As previously reported, this could be explained by the fluid retention as the pregnancy advances which leads to hemodilution that causes decrease in the sensitivity of a test [29]. The same findings were reported in a previous study whereby women with advanced pregnancy were found to have low human cytomegalovirus (HCMV) IgG titters as compared to their counterparts [30].

In the current study, it was found that being a housewife and being employed significantly predicted *B. melitensis* seropositivity. This could be explained by the fact that most of the urban residents with high socioeconomic status tend to consume grilled meat from common joints called “nyama choma” since they can afford the costs compared to their rural counterparts. Roasted/grilled goat meat has been reported to be the most common type of meat in urban settings [20]. Goat is the primary host for *B. melitensis*. Frequent consumption of roasted/grilled meat from these joints could lead to consumption of undercooked meat that can predispose them to *Brucella* spp. infections. Further studies to establish this relationship are warranted.

Having a history of miscarriage was also found to predict *B. melitensis* seropositivity. As previously reported, the markers of mixed infection of *B. melitensis* and *B. abortus* were found to be high among women with spontaneous abortion [31]. The same findings were also reported among women with spontaneous abortion in Saudi Arabia [32]. As opposed to animal species, it is believed that toxin production, bacteremia, fever, and disseminated intravascular coagulation play an important role in causing abortion in humans [10, 14, 33]. Further studies to investigate the role of *B. melitensis* in causing abortion in human are warranted.

This study could not establish active infection among pregnant women but the presence of *B. melitensis*-specific antibodies which might indicate past infections. Despite this limitation, this study has investigated a large sample size of pregnant women and documented the presence of *B. melitensis* antibodies and its associated factors.

## 5. Conclusion

Antibodies against *B. melitensis* were significantly high among employed and housewife pregnant women in urban settings of Mwanza. Furthermore, this study has also documented the association between gestation age, history of miscarriage, and *B. melitensis* seropositivity necessitating further prospective studies to explore these factors. There is a need to conduct more studies to establish the magnitude of brucellosis during early pregnancy among women presenting with signs and symptoms and follow these women to document pregnancy outcomes.

## Figures and Tables

**Table 1:** Sociodemographic characteristics of the enrolled women (*N* = 635).

Characteristics/variables		Frequency (*N*)	Percent (%)
Median (IQR) age (years)		25 (22-29)	
Median (IQR) gestation period (weeks)		21 (16-28)	
Median (IQR) parity		1 (0-3)	
Median (IQR) number of household members		4 (2-5)	
Location	Rural	86	13.5%
	Urban	549	86.5%
Religion	Christian	525	82.7%
	Muslims	110	17.3%
Marital status	Married	593	93.4%
	Single	42	6.6%
Education	Never attend school	53	8.3%
	Primary	366	57.6%
	Secondary	172	27.1%
	Tertiary	44	7%
Occupation	Employed	269	42.4%
	Housewives	178	28.0%
	Peasant	188	29.6%
Socioeconomic status (SES)	Low	128	20.2%
	High	507	79.8%
Keeping animals at home	Yes	47	7.4%
	No	588	92.6%

**Table 2:** Clinical characteristics of the participants (*N* = 635).

Characteristics/variables		Frequency (*N*)	Percent (%)
Body temperature^∗^		36.3°C (IQR:35.5-36.7)	
H/fever in current pregnancy	Yes	306	48.2%
	No	329	51.8%
Malaise	Yes	233	36.7%
	No	402	63.3%
Headache	Yes	328	51.7%
	No	307	48.3%
Myalgia	Yes	63	9.9%
	No	572	90.1%
Loss of appetite	Yes	204	32.1%
	No	431	67.9%
Eating raw meat	Yes	26	4.1%
	No	609	95.9%
Roasted meat	Yes	405	63.8%
	No	230	36.2%
H/baby with low birth weight	Yes	26	5.8%
	No	421	94.2%
H/miscarriage	Yes	72	11.3%
	No	563	88.7%
Stillbirth	Yes	12	2.7%
	No	435	97.3%
Blood transfusion	Yes	47	7.4%
	No	588	92.6%
HIV status	Positive	12	1.9%
	Negative	574	90.4%
	Unknown	49	7.7%

^∗^Median and interquartile range.

**Table 3:** Univariate and multivariate analysis of the factors associated with B. melitensis seropositivity.

Characteristics/variables		% positive	Univariate (OR, 95% CI)	*P* value	Multivariate (aOR, 95% CI)	*P* value
Age(years)		^∗^24 (22-29)	0.975 (0.938-1.013)	0.200	0.973 (0.931-1.016)	0.220
Gestation age (weeks)		^∗^20 (12-24)	0.954 (0.930-0.980)	0.001	0.972 (0.945-0.999)	0.045
Median (IQR) parity		^∗^1 (0-2)	0.885 (0.774-1.012)	0.075		
Number of household members		^∗^3 (2-4)	0.843 (0.698-1.018	0.077		
Location	Rural (86)	3 (3.5)	1			
	Urban (549)	100 (18.2)	6.161 (1.908-19.894)	0.002	0.626 (0.163-2.400)	0.495
Religion	Christian (525)	78 (14.9)	1			
	Muslim (110)	25 (22.7)	1.685 (1.015-2.797)	0.043	0.695 (0.407-1.187)	0.183
Marital status	Single (42)	3 (7.1)	1			
	Married (593)	100 (16.9)	2.637 (0.799-8.700)	0.111		
Education	Never Attended (53)	1 (1.9)	1			
	Primary (266)	60 (16.4)	10.196 (1.382-75.187)	0.023		
	Secondary (172)	33 (19.2)	12.345 (1.646-92.579)	0.014		
	Tertiary (44)	9 (20.4)	13.371 (1.621-110.286)	0.016		
Occupation	Peasant (188)	8(4.3)	1			
	Housewives (178)	41 (23.0)	6.733 (3.057-14.828)	≤0.001	3.902 (1.589-9.577)	0.003
	Employed (269)	54 (20.1)	5.651 (2.620-12.186)	≤0.001	3.405 (1.412-8.208)	0.006
Socioeconomic status (SES)	Low (128)	9 (7.0)	1			
	High (507)	94 (18.5)	3.009 (1.474-6.143)	0.002	1.336 (0.597-2.988)	0.480
Animal keeping	No (588)	98 (16.7)	1			
	Yes (47)	5 (16.6)	0.595 (0.229-1.542)	0.286		
History of fever	No (329)	56 (17.0)	1			
	Yes (306)	47 (15.4)	0.884 (0.579-1.350)	0.570		
Malaise	No (402)	59 (14.7)	1			
	Yes (233)	44 (18.9)	1.352 (0.881-2.780)	0.167		
Headache	No (307)	51 (16.6)	1			
	Yes (328)	52 (15.8)	0.945 (0.620-1.442)	0.796		
Myalgia	No (572)	88 (15.4)	1			
	Yes (63)	15 (23.8)	1.718 (0.922-3.203)	0.088		
Loss of appetite	No (431)	60 (13.9)	1			
	Yes (204)	43 (21.1)	1.651 (1.071-2.546)	0.023	1.567 (0.993-2.472)	0.053
Eating raw meat	No (609)	98 (16.1)	1			
	Yes (26)	5(19.2)	1.241 (0.457-3.371)	0.671		
Eating roasted meat	No (230)	28 (12.3)	1			
	Yes (405)	74 (18.5)	1.639 (1.026-2.618)	0.038	1.125 (0.680-1.862)	0.680
H/baby with low birth weight	No (609)	98 (16.1)	1			
	Yes (26)	5 (19.2)	1.241 (0.457-3.371)	0.671		
H/miscarriage	No (563)	84 (14.9)	1			
	Yes (72)	19 (26.4)	2.044 (1.152-3.625)	0.014	1.940 (1.043-3.606)	0.036
H/stillbirth	No (619)	101 (16.3)	1			
	Yes (16)	2 (12.5)	0.732 (0.163-3.273)	0.684		
H/blood transfusion	No (588)	94 (16.0)	1			
	Yes (47)	9 (19.2)	1.244 (0.582-2.659)	0.572		
HIV status	Negative (574)	84 (14.6)	1			
	Positive (12)	4 (33.3)	2.916 (0.859-9.902)	0.086	2.792 (0.745-10.467)	0.128
	Unknown (49)	15 (30.6)	0.573 (1.343-4.930)	0.004	1.658 (0.836-3.290)	0.147

^∗^Median with interquartile range.

## Data Availability

All data generated/analyzed during this study are included in this manuscript.
